# Randomized Controlled Trial of the ShangRing for Adult Medical Male Circumcision: Safety, Effectiveness, and Acceptability of Using 7 Versus 14 Device Sizes

**DOI:** 10.1097/QAI.0000000000001015

**Published:** 2016-05-24

**Authors:** Paul J. Feldblum, Robert Zulu, David Linyama, Sarah Long, Thikazi Jere Nonde, Jaim Jou Lai, Joshua Kashitala, Valentine Veena, Prisca Kasonde

**Affiliations:** * Global Health Department, FHI 360, Durham, NC;; † Department of Surgery, University Teaching Hospital, Lusaka, Zambia;; ‡ FHI 360, Lusaka, Zambia; and; § FHI 360, Nairobi, Kenya.

**Keywords:** male circumcision, ShangRing device, safety, Zambia, randomized controlled trial

## Abstract

**Objectives:**

To assess the safety, effectiveness, and acceptability of providing a reduced number of ShangRing sizes for adult voluntary medical male circumcision (VMMC) within routine service delivery in Lusaka, Zambia.

**Methods:**

We conducted a randomized controlled trial and enrolled 500 HIV-negative men aged 18–49 years at 3 clinics. Participants were randomized to 1 of 2 study arms (Standard Sizing arm vs Modified Sizing arm) in a 1:1 ratio. All 14 adult ShangRing sizes (40–26 mm inner diameter, each varying by 1 mm) were available in the Standard Sizing arm; the Modified Sizing arm used every other size (40, 38, 36, 34, 32, 30, 28 mm inner diameter). Each participant was scheduled for 2 follow-up visits: the removal visit (day 7 after placement) and the healing check visit (day 42 after placement), when they were evaluated for adverse events (AEs), pain, and healing.

**Results:**

Four hundred and ninety-six men comprised the analysis population, with 255 in the Standard Sizing arm and 241 in the Modified Sizing arm. Three men experienced a moderate or severe AEs (0.6%), including 2 in the Standard Sizing arm (0.8%) and 1 in the Modified Sizing arm (0.4%). 73.2% of participants were completely healed at the scheduled day 42 healing check visit, with similar percentages across study arms. Virtually all (99.6%) men, regardless of study arm, stated that they were very satisfied or satisfied with the appearance of their circumcised penis, and 98.6% stated that they would recommend ShangRing circumcision to family/friends.

**Conclusions:**

The moderate/severe AE rate was low and similar in the 2 study arms, suggesting that provision of one-half the number of adult device sizes is sufficient for safe service delivery. Effectiveness, time to healing, and acceptability were similar in the study arms. The simplicity of the ShangRing technique, and its relative speed, could facilitate VMMC program goals. In addition, sufficiency of fewer device sizes would simplify logistics and inventory.

## INTRODUCTION

Voluntary medical male circumcision (VMMC) provides enduring protection against HIV acquisition among men.^1–3^ The World Health Organization (WHO) and the Joint United Nations Programme on HIV/AIDS (UNAIDS) recommend that VMMC should be a key component in HIV prevention programs and set ambitious goals for adult and adolescent VMMC procedures in 14 sub-Saharan countries.^4^

The safety, effectiveness, and acceptability of VMMC procedures are clearly vital, as are program efficiencies to meet goals. Male circumcision (MC) devices have the potential to increase the number of clients reached with VMMC^5^ and create additional demand. Two MC devices have been prequalified by the WHO for use and surveillance under conditions of routine service delivery,^6,7^ including the ShangRing device from China.

ShangRing is a collar clamp circumcision device, consisting of 2 concentric plastic rings and a silicone-ringed pad that sandwiches the foreskin of the penis.^8^ The rapid, tight compression of the foreskin between the rings achieves hemostasis and prevents tissue slippage. Details of the ShangRing placement and removal procedures have been described elsewhere.^9^

At the time of the study, 14 sizes of ShangRing for adults were available, each size being 1 mm different than the next, selected by means of a special measuring tape placed around the penile shaft. The large number of adult ShangRing sizes poses an obvious procurement, distribution, and stocking challenge for VMMC programs. If use of fewer sizes was found to be equally safe and effective, supply logistics would be simplified. We conducted a randomized controlled trial (RCT) to assess the safety, effectiveness, and acceptability of having available every other ShangRing device size for adult VMMC. We aimed to provide an evidence base for policy decisions by officials considering integration of devices into national programs while seeking efficiencies that might be gained through simplified supply chains.

## METHODS

### Study Sites and Providers

The trial was conducted at 3 sites in Lusaka: the University Teaching Hospital (UTH) MC unit, Chilenje Clinic, and Chawama First Level District Hospital, all of which provide routine adult VMMC services. As with all VMMC methods, the ShangRing procedures were offered as part of the minimum package of HIV prevention services recommended by the Zambia Ministry of Health (MOH).

Providers, counselors, and health promoters at the clinics were trained on the ShangRing method, study procedures, completion of data collection forms, and postplacement, postremoval and HIV risk-reduction counseling.

### Inclusion/Exclusion Criteria

Inclusion criteria were the following:

- age 18–49 years;
- HIV-uninfected per same-day routine voluntary HIV testing at the clinic;
- uncircumcised on examination; and
- able to understand study procedures, agree to abide by them, voluntarily sign a written informed consent form, and provide full contact information including cell phone number.

Exclusion criteria were the following:

- active genital infection upon visual inspection;
- anatomic abnormality (eg, epispadias or hypospadias) that contraindicates ShangRing MC;
- penis is too big or small for available device sizes;
- takes a medication or has a self-reported medical condition that would be a contraindication for elective surgery;
- self-reported allergy or sensitivity to lidocaine or other local anesthesia; or
- cannot be circumcised on the same day as screening.

### Study Arms

We enrolled HIV-uninfected adult men seeking VMMC from October 2014 to July 2015. Participants were randomized to 1 of 2 study arms in a 1:1 ratio, using permuted blocks with randomly selected block sizes: the Standard Sizing arm, using the full array of 14 adult sizes (diameter of 26–40 mm varying by 1 mm each; the 27 mm device was unavailable); and the Modified Sizing arm, using only 7 adult sizes (diameter of 28–40 mm varying by 2 mm each). Sealed, numbered, opaque, tamper-evident envelopes were prepared and shipped to the study sites. FHI 360 study staff were blinded to the randomization sequence until the data set was frozen.

Providers were trained to measure penis circumference at the coronal sulcus using the measuring strip shipped with the devices. The exact millimeter measure was used for men in the Standard Sizing arm. In the Modified Sizing arm, we used the exact measurement if possible; if the measurement fell between 2 device sizes, the provider chose the smaller size.^10^

Each participant was scheduled for 2 follow-up study visits: the removal visit (day 7 after placement with an allowable window of day 5–9) and the healing check visit (day 42 after placement with an allowable window of day 40–44) although they were encouraged to return at any time with a complication or concern. Participants who were not completely healed at the day 42 visit were scheduled for additional visits at weekly intervals until complete healing was confirmed.

### Study Objectives and Endpoints

The primary objective of the trial was to assess the safety of ShangRing MC procedures when providers chose among 7 adult sizes (Modified Sizing arm) versus the full range of 14 adult sizes (Standard Sizing arm) during routine service delivery. The primary endpoint was the percentage [and 95% confidence interval (CI)] of men with at least 1 moderate or severe adverse event (AE) in each study arm. Although mild AEs were recorded, they were not included in the analysis endpoint nor reported here. AEs were classified according to a widely used consensus guide^11^ modified for MC devices and recorded at each visit on a dedicated case report form. We took digital penile photographs of AE cases, with separate consent of the participant. Photographs showed only the genital area, identified solely by the participant's study number and date of visit.

The secondary objectives of this research study were to:

- Assess the effectiveness of ShangRing procedures by measuring the percentage of men in each study arm who were successfully fitted and had a ShangRing placed;
- Determine the time to complete healing, generally at or after the healing check visit at day 42, with complete healing defined as no scab along the wound with dry and healthy appearance of the epithelium; and
- Evaluate the acceptability of ShangRing procedures among clients, including pain during and after the procedures on a scale of 0–10,^12^ and days needed to return to normal activities.

### Statistical Considerations

For the primary safety endpoint, with 250 men in each study arm, we had 80% power to detect an absolute difference in the proportion of moderate/severe AEs of at least 8 percentage points, using 2-sided tests at the 0.05 significance level for events observed in no more than 4% of the Standard Sizing (comparison) arm. The study had power to detect only large between-group differences in overall AE percentages. The study had approximately 80% power to detect any rare individual AE with a true rate of 0.008 (0.8%) or greater in each arm.

The full “Analysis Population” included all participants who underwent study screening and formed the population for baseline characteristics and study disposition. The “RCT Population” included the subset of eligible participants randomized to the 2 study arms and is the primary population for comparisons between arms. We conducted as-treated analyses in the RCT Population (ie, defining analysis groups according to the device size actually received, regardless of assignment errors). Missing data were assumed to be missing at random and ignored.

We tabulated the frequency and percentage of men with moderate or severe AEs in each study arm and overall, both pooled across sites and separately within sites. We computed the percentage of men with at least 1 moderate or severe AE and the 95% CI around the percentage in each arm as well as overall. The difference in percentages between the 2 RCT study arms (and 95% CI around the difference) was calculated using the method of Newcombe^13^ score with continuity correction.

We ascertained clinical features of the ShangRing procedures, particularly duration of device placement and removal procedures, and self-reported pain during the procedure, 30 minutes postprocedure, and during the 7-day wear period. Participant acceptability of the method, days to return to usual activities, and satisfaction with the postcircumcision cosmetic results were ascertained. We tabulated the percentage of men with complete healing at the scheduled day 42 follow-up visits.

### Ethical Considerations

We obtained ethical committee review and approval for the trial and subsequent protocol amendments from the FHI 360 and University of Zambia IRBs.

## RESULTS

### Baseline Features of the Cohort

Five hundred men met prescreening criteria and were enrolled, including 198 at UTH (39.6%), 155 at Chilenje (31.0%), and 147 at Chawama (29.4%). Upon physical examination to confirm eligibility, no one was screened out because his penis was too big or too small for the available sizes. Two men (0.4%) were excluded because of anatomical abnormalities (1 hypospadias; 1 small foreskin), one man withdrew after consent and enrollment, and one man was excluded from the analysis because of a consent error, leaving N = 496 men. Five men were randomly assigned to the Modified Sizing arm but erroneously received a size only available in the Standard Sizing arm; they were included in the Standard Sizing arm during analyses. A total of 255 men comprised the Standard Sizing study arm, and 241 men were included in the Modified Sizing arm.

The median age was 23.5 years [interquartile range (IQR) 20–29], was similar in the 2 study arms, and varied only slightly among the 3 clinics. The most prevalent ethnic group was Bemba, constituting 26.3% of the cohort overall (Table 1).

**TABLE 1.**
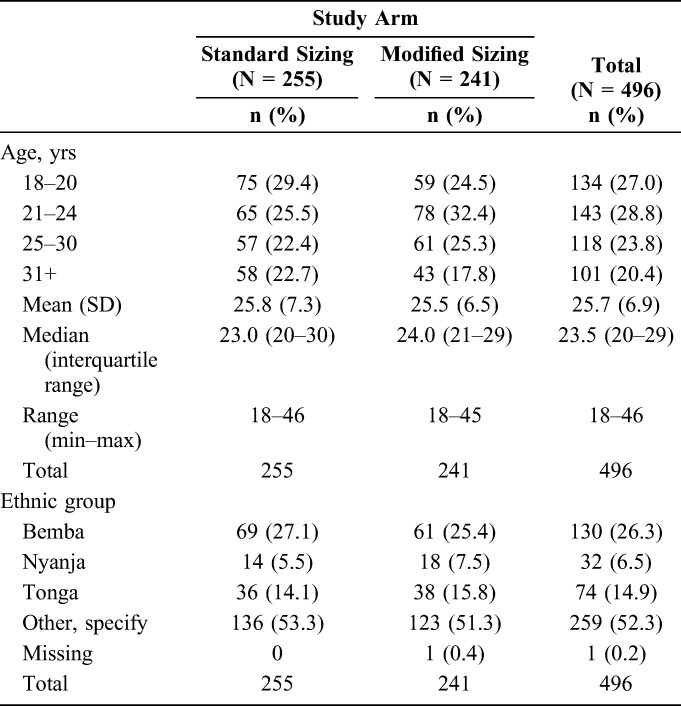
Demographic Features of Enrolled Participants, RCT Population

### Features of the Circumcision Procedures

The majority of the ShangRing procedures in both study arms was performed by nurses (92% in the Standard Sizing arm and 88% in the Modified Sizing arm). The median duration of placement was 8 minutes (IQR 6–10) in both study arms. One problem during placement was reported in each study arm, but neither event led to an AE. In the Standard Sizing arm, 5 of the 14 device sizes were each used for more than 10% of procedures (32–36 mm inner diameter). Median postoperative pain queried approximately 30 minutes after placement was 1 on a scale of 0-to-10 in both study arms.

The median duration of removal was 5 minutes in both study arms. Median removal pain was 3 in both study arms on the same 0-to-10 pain scale, falling to a median of 1 in both study arms immediately after removal.

### Adverse Events

Overall 3 men (0.6% of participants) experienced a moderate or severe AE (0.8% in the Standard Sizing arm and 0.4% in the Modified Sizing arm; Table 2). There were no statistically significant nor clinically meaningful differences in the overall AE rate, or the rates of specific types of AEs, between the 2 study arms.

**TABLE 2.**
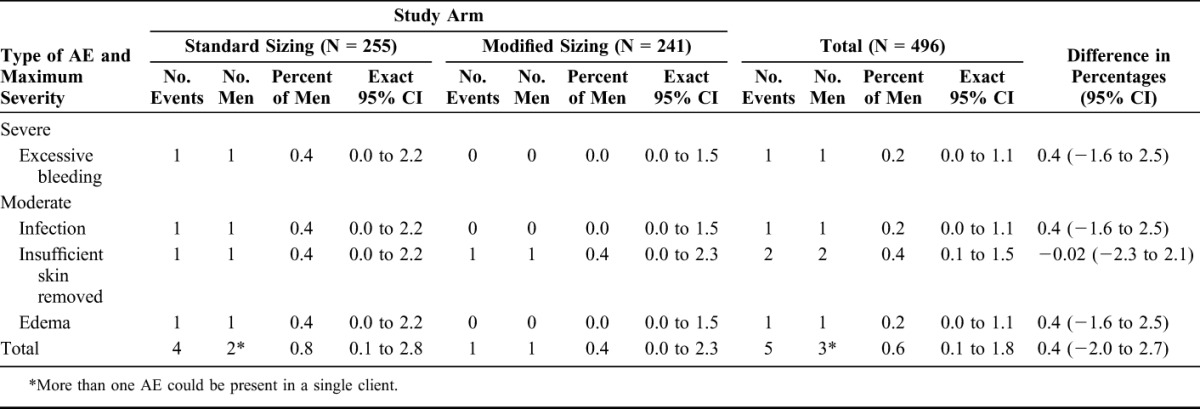
Moderate and Severe Adverse Events Related to ShangRing Circumcision, RCT Population

Two participants in the Standard Sizing arm had reportable moderate/severe AEs. The first had severe bleeding at day 12, 5 days after device removal. That event was designated a Serious AE (SAE) because it required suturing on 2 occasions before the bleeding could be stopped. Further tests of the man revealed a clotting disorder. The second participant had a moderate infection and moderate swelling at day 9 when the device was removed. At the day 42 healing check visit, he was also deemed to have moderate insufficient skin removal. The one participant in the Modified Sizing arm with a reportable AE had moderate insufficient skin removal diagnosed at the healing check visit. Neither participant with insufficient skin removal had decided on surgical completion of circumcision by the time of study exit.

### Study Status and Time to Healing

All participants who had a ShangRing placed returned for removal. Four hundred and eighty four participants (97.6%) were deemed completely healed at healing check visits although about a quarter of all healing check visits were after the scheduled day 40–44 visit window. Overall, 73.2% of all participants were completely healed at the day 42 healing check visit as scheduled (75.7% in the Standard Sizing arm and 70.5% in the Modified Sizing arm); 24.4% of all participants completed the study and were confirmed fully healed at a visit later than the scheduled day 42 visit (45 days or more after circumcision: 22.0% in the Standard Sizing arm and 27.0% in the Modified Sizing arm). Twelve men (2.4%) discontinued the study or were lost during follow-up before healing could be confirmed.

### Acceptability Parameters

Virtually all (99.6%) of the men stated that they were very satisfied or satisfied with the appearance of their circumcised penis, and 98.6% stated they would recommend ShangRing circumcision to a friend or family member. Of the many possible reasons for liking the procedure listed on our interview form, 5 reasons were each cited by more than 40% of participants in both study arms: quick procedure time; no stitches; less pain than expected; satisfactory cosmetic result; and improved personal hygiene. The form had the same number of possible reasons to dislike the procedure, and one reason was cited by 25% of participants in both study arms: pain or discomfort with erection during the 7-day device wear period. However, half of participants overall (52.5%) did not report a single reason to dislike the procedure. Frequencies of these acceptability factors differed little between the study arms.

Participants were queried about how much the pain interfered with walking, sleep, and work. Such interference was deemed mild on average by men in both study arms and tended to diminish during the 7 days of device wear. More than 80% of men in each study arm returned to normal activities within 2 days of the circumcision procedure. Maximum pain during erection at any time during device wearing was moderate in both study arms: median 4 on the scale of 0-to-10.

## DISCUSSION

Our trial conducted in Lusaka, Zambia, observed few moderate/severe AEs. The AE rate was low and equivalent in the 2 study arms, and healing times were comparable, indicating that provision of one-half the number of adult device sizes is sufficient for safe service delivery of the ShangRing.

The benefit of proper ShangRing device sizing for circumcision safety and comfort seems obvious. But in practice, the penile measurement process is imprecise and somewhat subjective. On the one hand, the measuring tape can be squeezed slightly, thereby diminishing the reading; on the other hand, slight tumescence during measurement can increase circumference. And men can measure in between the available device sizes. A small RCT (N = 74) in China enrolled men with penile measurements between the available device sizes and assigned half to receive the next smaller device size and half to receive the next larger size.^10^ Selection of the smaller size resulted in less mean blood loss and edema, quicker removal times, and faster healing. Smaller rings made eversion of the foreskin easier, and dorsal slits were necessary to allow foreskin eversion in more men in the larger ring size study arm. The size of the trial resulted in low power to detect differences in these study outcomes, however. The current larger trial showed that availability of half the adult device sizes did not reduce safety, effectiveness, or acceptability.

Our results agree with other ShangRing data from African settings. Three other large ShangRing clinical studies have been conducted in Zambia, Kenya and Uganda,^9^ in which moderate/severe AE rates were 7.6%, 1.0%, and 1.6%. The concerning 7.6% figure emerged in the initial RCT of the device compared with conventional surgical circumcision in Kenya and Zambia; the AE rate in the surgery study arm was also unusually high in that trial (5.0%).^14^ A subsequent larger field study in the same 2 countries observed a much lower AE rate of 1.6%.^15^ Short-term outcomes of ShangRing circumcision compare favorably with conventional surgical procedures,^16^ and 2 studies with longer-term follow-up of men circumcised with the ShangRing device found few complications 2–3 years after the procedures.^17,18^

Only 2 of 500 men enrolled in this trial were found to be unsuitable for the ShangRing, an effectiveness rate of 99.6% (screen-out rate 0.4%). The ShangRing procedure could not be completed for 1 of 200 men in the earlier RCT (0.5%); 4 of 508 men in the Uganda study (0.8%); and 5 of 1211 men in the field study in Kenya and Zambia (0.4%).^9^ Thus far, it seems that only a small percentage of men will be anatomically unsuitable for the ShangRing procedure.

When queried about various aspects of circumcision procedure acceptability, trial participants revealed very favorable opinions about the ShangRing. In these dimensions, our study participants resembled those in previous ShangRing studies in Africa.^9^ One might expect differences between the study arms in self-reported pain and discomfort during device wear, but the pain score distributions were quite similar across arms immediately postplacement, during device wear, and at removal.

One strength of clinical research on the ShangRing is that under current guidelines, men are required to return for device removal at 7 days after circumcision. All participants in this study complied, so that it is unlikely that we failed to ascertain early AEs (within 7 days) and doubtful that our observed AE rate, the primary study endpoint, was biased downward.^19^

A weakness of this study is that almost one quarter of our study participants completed their healing check visits after the scheduled visit window of 40–44 days, biasing the measurement of healing times. We know that the percentage completely healed was at least 75% in the Standard Sizing arm and 70% in the Modified Sizing arm, both of which are lower than what is typically observed following surgical circumcision.^20^ The trial reaffirms healing times 1–2 weeks longer after device circumcisions than surgical procedures.^8^

The clinical profile of the ShangRing device for adult VMMC in sub-Saharan Africa continues to be favorable and is not affected by an approach that uses fewer device sizes. In addition to this study, other operational research on ShangRing is underway. A modification of the ShangRing circumcision procedure, termed the “no-flip” technique because it omits the foreskin eversion step, makes it even simpler to learn and apply in programs.^21^ A study of the safety of this technique and the acceptability of topical anesthesia for it is underway in Kenya (Barone M, personal communication). If VMMC demand can be increased by means of community-based interventions,^22^ the ease with which different cadres of providers can learn to safely and effectively apply the ShangRing technique, its relative speed, and the high acceptability consistently reported across studies could facilitate achievement of program goals for male circumcision.
